# Effects of Yangxinshi tablet on exercise tolerance in patients with coronary heart disease: A protocol of randomized, double-blind, placebo-controlled, and multi-center trial

**DOI:** 10.1097/MD.0000000000021485

**Published:** 2020-07-31

**Authors:** Sisi Zhang, Congying Liang, Yang Yang, Zhijia Zhao, Jiaojiao Li, Xiaoping Meng

**Affiliations:** a Cardiovascular and Cardiac Rehabilitation Department, First Affiliated Hospital of Changchun Chinese Medicine University, Changchun City, Jilin Province; b Operating Room Department, Feicheng Mining Bureau Central Hospital, Tai’an City, Shandong Province, China.

**Keywords:** cardiac rehabilitation, coronary heart disease, exercise tolerance, traditional Chinese medicine, Yangxinshi tablet

## Abstract

**Background:**

Exercise intolerance is very common in patients with coronary heart disease (CHD). Although some researches confirming the validation of traditional Chinese medicine (TCM) on CHD treatment, the effect of TCM on improving the exercise tolerance of patients with CHD remains unclear so far. Our trial is to investigate whether the Yangxinshi (YXS) tablet can improve exercise tolerance as well as the quality of life among CHD patients.

**Methods:**

It is a randomized, double-blind, placebo-controlled, multi-center trial. A total of 90 patients with CHD from 3 hospitals in China will be enrolled and randomly assigned to one of 2 groups: YXS group, N = 45; placebo group, N = 45. The 2 groups will simultaneously receive standardized western medicine and exercise-based cardiac rehabilitation program for 12 weeks. The primary outcome measure is the exercise capacity, which will be evaluated by the cardiopulmonary exercise test and 6-minute walking test. The 2nd outcomes include symptom improvement, psychologic issues, laboratory tests, side effects, and adverse events.

**Discussion:**

To our knowledge, it is the 1st randomized controlled trial to evaluate the effect of TCM YXS tablet on exercise tolerance in patients with CHD. The results will provide more evidence for future studies in this area.

**Trial registration:**

This study protocol was registered in Research Registry (researchregistry5752).

## 1 Introduction

Cardiovascular disease, especially coronary heart disease (CHD), has been a major threat to human life,^[1–3]^ whose morbidity and mortality have been increasing over the past 30 years in China. It is responsible for one-third of all deaths worldwide, and its incidence is forecasted to increase steadily, which will impose a huge health and financial burden on the whole country.^[4–6]^ Exercise capacity (EC), also called exercise tolerance, refers to the maximum aerobic capacity that can be sustained without the occurrence of pathologic symptoms and/or medical signs, which serves as a strong predictor of cardiovascular and all-cause mortality.^[7,8]^ As reports, the increase of average 1.0 metabolic equivalent (MET; 1 MET = 3.5 mL/kg/min) will be accompanied by the 12% improvement of survival.^[8]^ Peak oxygen consumption (VO_2_ peak) or MET can be estimated or directly measured by cardiopulmonary exercise test (CPET) or 6-minute walking test (6MWT). Exercise intolerance, characterized by dyspnea or fatigue, is an independent predictive factor and associated with poor prognosis in patients with CHD. As a multidisciplinary and comprehensive program, exercise-based cardiac rehabilitation (CR) can effectively improve EC, the quality of life (QoL) in patients with CHD. It also has other potential benefits including decrease ischemia, improves endothelial function, subsequently reduces readmissions, mortality, and morbidity.^[9–11]^ American Heart Association (AHA)/American College of Cardiology (ACC) recommend CR as a class Ia for patients with acute myocardial infarction or coronary revascularization.^[12]^ From the perspective of traditional Chinese medicine (TCM), the pathogenesis of CHD is Qi and Yin deficiency, also named as “thoracic obstruction” or “cardialgia.”^[13]^ TCM, as a unique medical formulate, has been used in China for more than 2000 years and plays an important role in the treatment of cardiovascular diseases.^[14]^

Yangxinshi (YXS) tablet, manufactured by Qingdao Growful Pharmaceutical Co, Ltd (Qingdao, China), is composed of thirteen kinds of Chinese herbal medicines, its active ingredient ginseng is considered as a supplement for Qi. It has been widely used for the treatment of CHD, relieving symptoms of angina pectoris, and protecting myocardial ischemia.^[15]^ A previous study has already reported the effects of promoting rehabilitation after the percutaneous coronary intervention with no obvious side effect,^[16]^ whether it could help improve EC for patients with CHD is still unclear. Our study aims to investigate the efficacy and safety of YXS tablet on the exercise tolerance in CHD and its role in the CR program.

## 2 Methods

### 2.1 Objective

The objective of this study is to assess the efficacy and safety of YXS tablet on improving the exercise tolerance of patients with CHD, to provide evidence for its application in the CR program.

### 2.2 Study design

**Figure 1:**
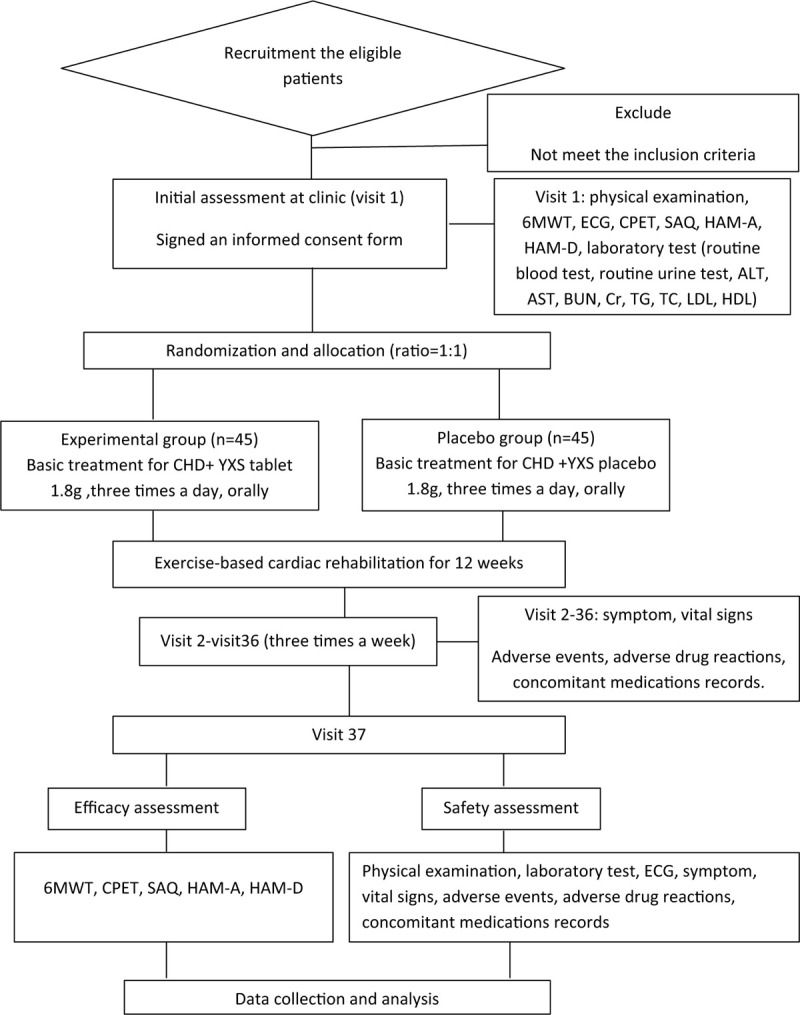
The flowchart of the trial. ALT = alanine aminotransferase, AST = aspartate aminotransferase, BUN = blood urea nitrogen, CHD = coronary heart disease, CPET = cardiopulmonary exercise test, Cr = creatinine, ECG = electrocardiograph, HAM-A = Hamilton anxiety rating scale, HAM-D = Hamilton depression rating scale, HDL = high-density lipoprotein, LDL = low-density lipoprotein, 6MWT = 6-minute walking test, SAQ = Seattle Angina questionnaire, TC = total cholesterol, TG = triglyceride, YXS = Yangxinshi.

This study is a randomized, double-blind, placebo-controlled trial, which has been registered in Research Registry (researchregistry5752). The specific flowchart is shown in Figure 1. All the eligible patients will be randomly assigned to one of the 2 groups: experiment group (basic medicine for CHD + YXS tablet) and control group (basic medicine for CHD + YXS placebo tablet) at the ratio of 1:1. This trial adheres to the Recommendations for Interventional trials of the standard protocol items (SPIRIT) 2013 statement.^[17]^

### 2.3 Ethical issues

This study is following with the Declaration of Helsinki and been approved by the institutional ethics committee of the first affiliated hospital of Changchun Chinese Medicine University, with the ethics number CCZYFYLL2017-038. It is also performed at Tongji hospital affiliated to Shanghai Tongji University, and Jinqiu Hospital in Liaoning Province. Any revision of protocol will be submitted to these ethics committees.

### 2.4 Participants

Patients will be recruited from three hospitals. As the leader unit of this trial, we began to recruit the patients from the outpatient or inpatient in March 2018. Specialists will explain the purposes, methods, benefits, and potential side effects of this study, then all participants need to sign an informed consent form.

### 2.5 Criteria

#### 2.5.1 Inclusion criteria

1. Aged 40 to 75, with no sex limitation.
2. History of CHD, with or without revascularization treatment.
3. Coronary angiography or coronary computed tomography angiography shows at least 1 major vessel's luminal diameter stenosis >50% according to the guidelines of western medicine diagnostic criteria of CHD.^[18,19]^
4. The patient is in a stable state, without emergency issues in recent 3 months.
5. Completely understand the significance of this study and volunteer to participate.
6. The patient agrees to follow all the procedures of this study and sign informed consent.

#### 2.5.2 Exclusion criteria

1. Have a history of the acute coronary syndrome over the past 3 months.
2. Uncontrolled hypertension (systolic blood pressure ≥180 mm Hg and/or diastolic blood pressure ≥100 mm Hg) or hypotension (systolic blood pressure <90 mm Hg and/or diastolic blood pressure <60 mm Hg).
3. Having a history of stroke (cerebral hemorrhage, subarachnoid hemorrhage, cerebral thrombosis, cerebral embolism) or lower extremity peripheral arterial disease over the past 6 months.
4. Pregnancy, breastfeeding, preparing for pregnancy, and perinatal female.
5. With episode bronchial asthma, chronic obstructive pulmonary disease, bradycardia (resting heart rate <60 bpm), cardiac pacemaker, or atrioventricular block.
6. Severe allergic constitution, known or likely to be allergic to the test drug or its components.
7. Known bleeding tendency or hemorrhagic disease.
8. Patients with serious liver and kidney dysfunction (creatinine clearance ≤40 mL/min or in the active stage of kidney disease, serum aminotransferase ≥2 × upper limit of clinical reference), other life-threatening serious primary or psychiatric diseases, and malignant tumors.
9. Patients participated in other clinical trials during the past 3 months.
10. Any other situations which may affect the outcomes of the trial.

### 2.6 Withdrawal and drop-out

#### 2.6.1 Withdrawal and drop-out decided by the investigator

The investigator's decision to withdraw refers to the cases in which it is not appropriate to continue the trial, including the following conditions:

1. Patients who have used the medicine for more than 4 weeks should be included in the efficacy statistics; discontinuation or conversion of other similar medicines after 4 weeks (including 4 weeks) should be treated as ineffective and need to be dropout;
2. During the trial, patients should be withdrawn and treated as invalid if they developed myocardial infarction or angina pectoris, which could not be relieved by nitroglycerin.
3. During the trial, any changes of condition which will affect the compliance and the safety of the patients can be decided to discontinue by the investigator.
4. The patients seriously violate the study scheme, have poor compliance with the trial, or consume the prohibited drugs.

#### 2.6.2 Withdrawal and drop-out decided by patients

All the patients are allowed to withdraw from the trial at any time without any reason. In some cases, patients no longer adapt the medical treatment and refuse to be followed up, even though they do not explicitly withdraw, can also be classified as “withdrawn.” For the cases who withdraw during the trial, the investigators carefully record the reasons, the medication process, and the last medication time. For those who withdraw or fail to come to the hospital on time, the investigators should try to inquire about the reasons via phone or letter, record the medication process, as well as the last medication time in detail. The relationship between withdrawal and the trial shall be carefully analyzed and recorded in the case report form (CRF). All original materials should be preserved.

### 2.7 Discontinuation conditions

Reasons for discontinuation include but not limited to the following circumstances:

1. the patient needs to hospitalize for revascularization therapy;
2. occurrence of serious adverse events (AEs) related to experimental drugs;
3. occurrence of serious safety issues;
4. financial or management reasons;
5. administrative authorities.

Once the discontinuation occurs, the safety and rights of the subject shall be protected as far as possible. The researchers or other staff shall cooperate in the following area: submit all generated research data; resolve all data problems; calculate, check and arrange all unused research products; and review the completeness of the research records.

### 2.8 Study procedure

**Figure 2:**
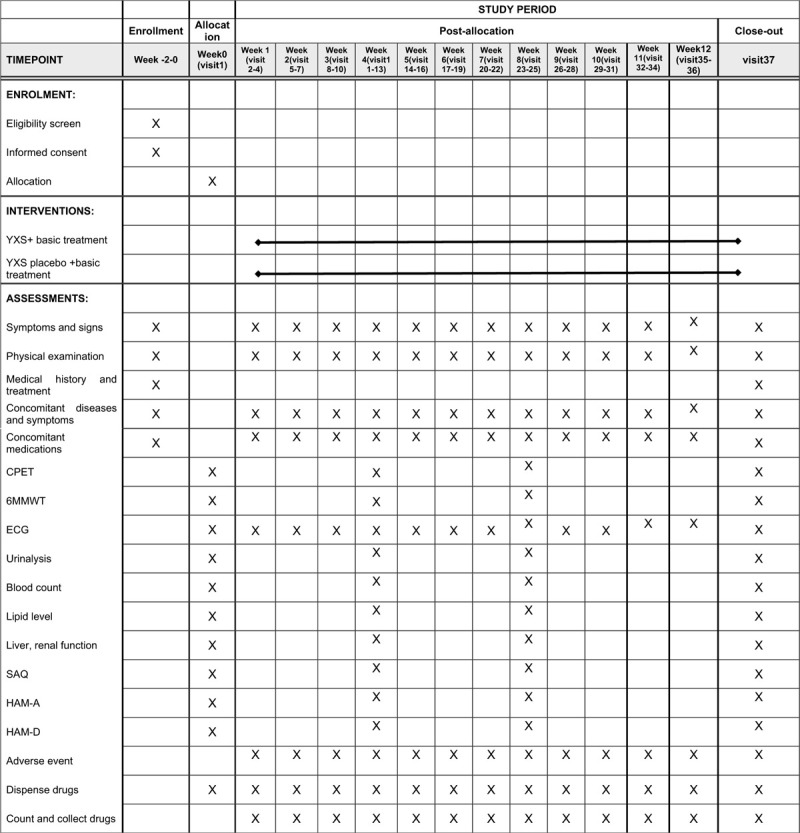
Schedule of enrollment, interventions, and assessments.

After signing informed consent, eligible participants will be randomized into the YXS group and placebo group. An investigator carries out the baseline evaluation, named visit 1, which contain the basic information, medical history, physical examination, CPET, 6MWT, electrocardiograph (ECG), Seattle Angina Questionnaire (SAQ), Hamilton anxiety rating scale (HAM-A), Hamilton depression rating scale (HAM-D), laboratory tests, etc. Visits including symptoms, vital signs, physical examination, ECG, concomitant medications and AE will be performed 3 times per week for the following 12 weeks. At the end of the trial, the same evaluations will be performed as the visit one. The schedule of enrollment, visits, and assessments is shown in Figure 2.

#### 2.8.1 CPET (from baseline to 12 weeks)

We adapt the symptom-limited CPET on a bicycle ergometer with the Bruce protocol. The initial workload is 5 W with loadless pedaling and increased 10 W every minute. The pedaling rate of 60 r/min is used to maintain consistency. The test discontinues until patients reach their limit of tolerance, such as complaining of fatigue, chest pain, shortness of breath, dyspnea, or abnormal ECG records are observed. At the end of the test, patients continue cycling with free-wheeling for 3 to 5 minutes, measurements will be continued during this period. Following parameters obtained from this test: peak VO_2_, ventilation (VE), carbon dioxide production (VCO_2_), ventilatory slopes for carbon dioxide production (VE/VCO_2_), respiratory exchange ratio, oxygen pulse, anaerobic threshold, using V-slop method,^[20]^ maximum ventilation (VE_max_), and peak workload. This test is performed and supervised by the trained staff.

#### 2.8.2 6MWT (from baseline to 12 weeks)

This test will be performed under the American Thoracic Society guidelines.^[21]^ Participants will be instructed to walk at their peace as far as possible along a flat, straight corridor, at least 50 m with a hard surface. The distance in 6 minutes will be recorded by the staff.

#### 2.8.3 SAQ (from baseline to 12 weeks)

The SAQ is designed to assess the disease-specific health status related to CAD. It contains 19 items, with the score ranges from 0 to 100, in which higher scores indicate better health status.^[22]^

#### 2.8.4 HAM-A (from baseline to 12 weeks)

The HAM-A is designed to assess the severity of anxiety, which contains 14 items related to a 5-point scale, with the score ranges from 0 to 4.^[23]^

#### 2.8.5 HAM-D (from baseline to 12 weeks)

The HAM-D is widely adopted in clinical trials assessing the effectiveness of antidepressants in the past decades. It consists of 17 items, which designed to measure the participants’ depressive symptomology.^[24]^

To avoid the risk of subjective influence, all these questionnaires performed and recorded by the same investigator at the baseline and 12 weeks.

### 2.9 Intervention

#### 2.9.1 Basic treatment

All the participants continue the conventional western medical treatment for CHD, such as antiplatelet, β-receptor blockers, angiotensin-converting enzyme inhibitors, angiotensin receptor inhibitors, statins, and nitroglycerin. Other kinds of medicines that would not affect the results, such as antibiotics, anti-inflammatory drugs, vitamins, laxatives, hypnotics, etc, can also be allowed to be taken during the trial. Moreover, information about these medicines or concomitant treatments must be recorded in the CRF for future analysis.

#### 2.9.2 Prohibited medicine

The following medicine including trimetazidine, any other TCM except for YXS tablet, antiarrhythmic, amiodarone, digitalis, β_2_-receptor agonist, tricyclic antidepressants, benzyprodil,pixicillin, sildenafil, vardenafil, and similar medicines are prohibited during the trial. If the patients’ condition gets worsen and has to take these above medicines, the case should be regarded as withdrawal or drop-out, and need to be recorded in the CRF.

#### 2.9.3 Experimental intervention

About 1.8 g of YXS tablet (0.3 g/tablet) will be taken in the experimental group, 3 times a day, orally, while 1.8 g of placebo will be taken in the control group with the same usage both for 12 successive weeks. YXS tablet is manufacture by Qingdao Growful Pharmaceutical Co Ltd. The main components are list in Table 1.^[25]^ The control group receives placebo treatment, which mainly consists of starch without any effective ingredient. The placebo is provided by the same manufacturer as YXS. It is a dextrin that matches as much as possible in appearance, shape, color, size, package, and the taste with YXS table with no side effects on human health. All these medicines are consistent with the Chinese Medicine Standards of the State Food and Drug Administration.

**Table 1:**
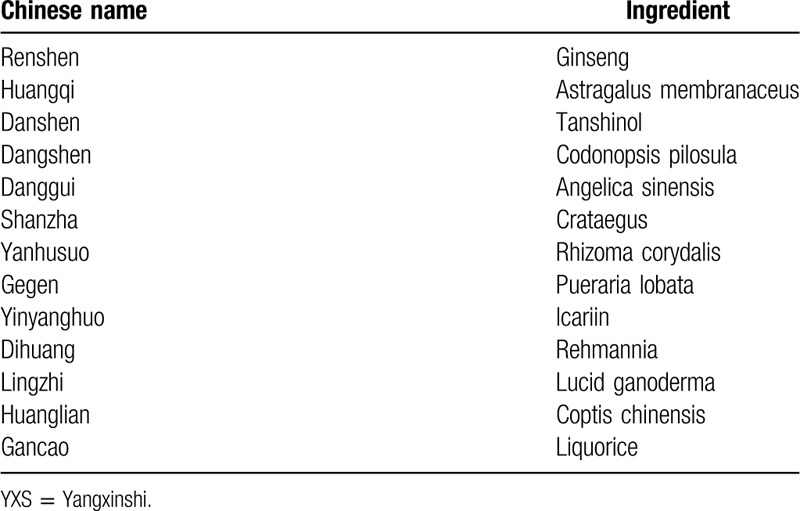
Main components of YXS table.

#### 2.9.4 Exercise intervention

All the patients participate in the aerobic exercise for 12 weeks (3 times weekly) performed in the outpatients with no time limitation. Each session last 30 minutes, which includes 5 minutes warm-up, 20 minutes aerobic exercise on a cycle ergometer or treadmill, and a 5 minutes cool-down according to the exercise-based guideline in CR.^[26]^ The training intensity is determined based on the heart rate recorded at the anaerobic threshold assessed by CPET to customize a personalized exercise prescription. The detailed exercise prescription is shown in Table 2. Both the 2 groups are in the same facility, with an equal level of prescription and supervision.

**Table 2:**
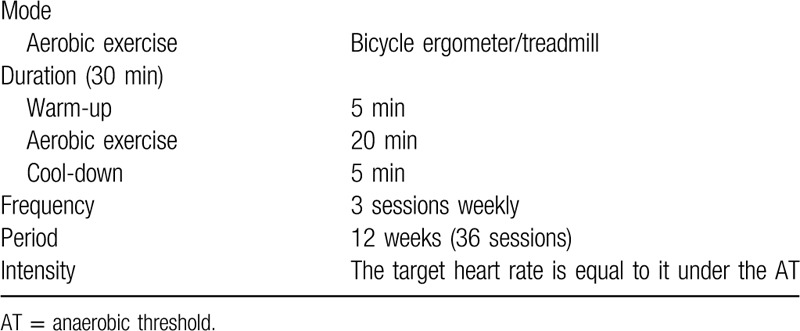
Exercise prescription.

### 2.10 Compliance and adherence

Compliance and adherence have a significant influence on the outcomes of CR. To ensure the compliance of exercise, the investigator checks the CRF weekly with medical and exercise performed, and makes telephone calls follow-up if necessary.

At each visit time point, patients are also asked to return the remaining drugs; thus, the investigator calculates the drug compliance with the following formula: 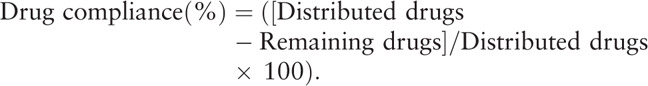


### 2.11 Randomization and blinding

The randomization sequences of the population and drug are generated by the computer-based software program. The drugs are distributed according to the serial number, and the patients are randomly allocated (as 1:1 ratio) to the experimental or control group. Drug distribution is supervised by the main research coordinator, who had no role in the evaluation and management, is also responsible for supplying enough materials and inventories timely for each center. Randomization sequence and intervention allocation will be kept in the Qingdao Growful Pharmaceutical Co Ltd so as it can be unblinded when necessary.

It is a double-blinded clinical trial, patients, investigators, and statistical staffs involved in are unaware of the treatment allocations until the study is completed in all the research centers.

### 2.12 Sample size

The sample size calculation was based on the primary endpoint (EC). According to the previous report, the mean value of VO_2_ peak in the experimental group is estimated to be 20 mL/kg/min, while that in the control group is 18.5 mL/kg/min.^[27]^ The sample size is calculated using the PASS 15.0 software. A sample size of 39 achieving 90.487% power is sufficient to reject the null hypothesis, indicating that when the mean difference in population is 1.5 mL/kg/min, a standard deviation for both groups would be 2.0 mL/kg/min, with a significance level (*a*) of 0.05. Allowing for the shedding rate of 10% to 20%, a total of 90 cases need to be enrolled as a type I error rate of *a* = 0.05, a power of 80%, with each center responsible for 30 cases.

This calculator uses the following formulas to calculate sample size and power, respectively: 
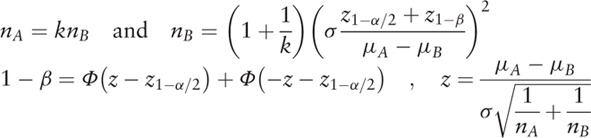


### 2.13 Statistical analysis

Statistical analysis will be performed by the Statistical Analysis System (SAS) software (SAS Institute Inc, Cary, NC). Continuous variables will be presented as the mean ± standard deviation, along with the analysis of the *t* test. Categorical variables will be presented as percentages and analyzed by the Chi-squared test. Variance analysis will be used to compare the changes in the SAQ, HAM-A, and HAM-D scores between the YXS group and the placebo group from baseline to 12 weeks. All tests are 2-sided tests and *P* < .05 is considered statistically significant. Intent-to-treat analysis of effectiveness and safety will also be carried out.

### 2.14 Data collection and management

Data will be collected and recorded by the investigator on the CRF at each visit time points (a total of 37). Each center securely stores the data in its computer system. The data is anonymous, only the main investigator has access to the data. Once the trial is completed, all the data will be frozen respectively. All the co-investigators are responsible for data monitoring.

### 2.15 Outcome measures

#### 2.15.1 Primary outcome

The primary outcome of this trial is EC. Changes of the VO_2_ peak and 6MWT from baseline to 12 weeks between 2 groups will be compared.

#### 2.15.2 Secondary outcome

The secondary outcome includes left ventricular ejection fraction, lipid level (triglyceride, total cholesterol, low-density lipoprotein, high-density lipoprotein), SAQ, HAM-A, and HAM-D scores.

### 2.16 Safety assessment

Symptoms, vital sign (temperature, blood pressure, breathing, and heart rate), physical examination, blood routine (red blood cell, white blood cell, hemoglobin, platelet), urine routine, liver and renal function (alanine aminotransferase, aspartate aminotransferase, blood urea nitrogen, creatinine), and ECG will be performed for consideration of safety.

### 2.17 Adverse event

Treatment-related AE will be assessed by CTCAE4.0 at each visit.^[28]^ The supervisor will carefully record all the details, such as time of occurrence, degree of AE, and probable causes on CRF. Once AE occurs, researchers should unblind and provide optimal treatment immediately. Any AE need to be submitted to the ethics committee within 24 hours.

## 3 Discussion

On the base of exercise-based CR, whether the YXS tablet combined with basic treatment can improve exercise tolerance as well as the QoL in patients with CHD will be investigated in this trial. To our best knowledge, this is the first randomized controlled trial evaluating the effects of TCM in the CR program; hence, the outcomes will provide the evidence for future, large-scale, clinical trials.

According to traditional Chinese theory, Qi, as the most basic substance constituting and sustaining the human body, could activate blood circulation, remove blood stasis, and relieve painfulness.^[29]^ For CHD patients, Qi deficiency and blood stasis are the main pathogenesis, which further limits EC.^[30,31]^ The unbalance between oxygen delivery and demand response for exercise intolerance.^[32]^ Although traditional Chinese methods, such as Taiji and Ba Duanjin, have been adopted in the exercise prescription, Chinese herbs still lack enough evidence. As a certified Chinese patent medicine developed and manufactured by Shanghai Pharmaceuticals Holding Co Ltd, YXS tablet contains various complicated components that play an important role in supplementing Qi, such as Astragalus membranaceus, rhizoma anemarrhenae, rhizoma corydalis, etc. It has been illustrated in previous researches that YXS could benefit rehabilitation after percutaneous coronary intervention and myocardial ischemia.^[16,33]^ We speculate that the active compounds of YXS for supplying Qi could improve myocardial energy metabolism, further improve the EC for CHD patients, this still requires confirmation by evidence-based clinical trials.

The EC serves as the primary endpoint in the study, and SAQ, HAM-A, HAM-D are also observed. CR as a multidisciplinary and promising program can also effectively relieve depression and anxiety, thus improve QoL in patients with CHD.^[34–36]^ Previous studies reported the effects of YXS on improving depression-like behaviors,^[15]^ its role in CR still needs further investigation. The safety of the YXS tablet will be evaluated as patients’ symptoms and laboratory tests, such as blood routine, urine routine, liver and renal function, and echocardiography.

To improve the validity and representativeness of the samples and then reduce the risk of selection bias, 3 comprehensive 3rd-grade 1st-class hospitals in mainland China participate. This study is a double-blind, randomized, placebo-controlled clinical trial, which will provide higher quality evidence regarding YXS application in the CR program.

## Acknowledgment

The authors thank Tongji hospital affiliated to Shanghai Tongji University, and Jinqiu Hospital in Liaoning Province for participating.

## Author contributions

**Conception and design:** Sisi Zhang, Xiaoping Meng

**Data curation:** Zhijia Zhao.

**Manuscript writing:** Sisi Zhang

**Methodology:** Jiaojiao Li.

**Recruiting the participants:** Sisi Zhang, and Yang Yang

**Review and editing:** Xiaoping Meng

**Supervision and coordination:** Congying Liang, Zhijia Zhao, Jiaojiao Li

**Supervision:** Congying Liang.

**Validation:** Yang Yang.

**Writing – review & editing:** Xiaoping Meng.
